# Algebraic overshear density property

**DOI:** 10.1007/s13366-023-00729-4

**Published:** 2024-01-02

**Authors:** Rafael B. Andrist, Frank Kutzschebauch

**Affiliations:** 1 Department of Mathematics, American University of Beirut, Beirut, Lebanon; 2 Faculty of Mathematics and Physics, University of Ljubljana, Ljubljana, Slovenia; 3 Mathematical Institute, University of Bern, Bern, Switzerland

**Keywords:** Shear, Overshear, Locally nilpotent derivation, Density property, Flexibility, Holomorphic automorphism, Bordered Riemann surface, Embedding of Riemann surfaces, Primary 32M17, 14R10, Secondary 32Q56

## Abstract

We introduce the notion of the algebraic overshear density property which implies both the algebraic notion of flexibility and the holomorphic notion of the density property. We investigate basic consequences of this stronger property, and propose further research directions in this borderland between affine algebraic geometry and elliptic holomorphic geometry. As an application, we show that any smoothly bordered Riemann surface with finitely many boundary components that is embedded in a complex affine surface with the algebraic overshear density property admits a proper holomorphic embedding.

## Introduction

### History

Starting with the seminal work of Andersén and Lempert (1992) on $\mathbb {C}^n, \, n \ge 2,$ the study of complex manifolds with infinite-dimensional holomorphic automorphism groups has been an extremely active area in several complex variables. At the same time, the study of such highly symmetric objects in affine algebraic geometry has been very active as well. In fact, the study of the algebraic automorphism group of $\mathbb {C}^n$ has started much earlier than in several complex variables. The starting point of the complex analytic investigations has been motivated by results and questions from the algebraic case. For example, Rosay and Rudin (1988, Open Question 6, p. 79) asked in 1988 whether the group of (volume-preserving) holomorphic automorphisms of $\mathbb {C}^n$ was generated by shears in coordinate directions, i.e., maps of the form

1 $$\begin{aligned} (z_1, \ldots , z_n) \mapsto (z_1, \ldots , z_{n-1}, z_n + f (z_1, \ldots , z_{n-1})), \end{aligned}$$

where $f \in \mathcal {O}(\mathbb {C}^{n-1})$ is an arbitrary polynomial or holomorphic function of $n-1$ variables. They can be viewed as time-1 maps of the vector field $\theta = f(z_1, \ldots , z_{n-1}) \frac{\partial }{\partial z_n}$. In complex analysis (when *f* is holomorphic) such an automorphism is called a shear and such a vector field is called a shear field. If *f* is a polynomial, the complex analysts call the automorphism a polynomial shear, whereas in affine algebraic geometry it is called an elementary automorphism.

Overshears in coordinate directions are maps of the form

2 $$\begin{aligned} (z_1, \ldots , z_n) \mapsto (z_1, \ldots , z_{n-1}, f (z_1, \ldots , z_{n-1}) \cdot z_n), \end{aligned}$$

where $f \in \mathcal {O}^*(\mathbb {C}^{n-1})$ is a nowhere vanishing holomorphic function. By simple connectedness of $\mathbb {C}^{n-1}$, the function *f* is the exponential $f = e^g$ of some holomorphic $g \in \mathcal {O}(\mathbb {C}^{n-1})$. Again, such an automorphism is the time-1 map of a complete(ly integrable) holomorphic vector field $\theta = g(z_1, \ldots , z_{n-1}) z_n \frac{\partial }{\partial z_n}$.

The problem in affine algebraic geometry that corresponds to the question of Rosay–Rudin, is the *tame generator conjecture*, asking whether polynomial maps of the form (1) together with affine automorphisms (the group generated by them is called the tame subgroup) generate the algebraic automorphism group of $\mathbb {C}^n$. This is classically known to be true for $n=2$, much later it has been shown by Shestakov and Umirbaev (2003) to be wrong for $n=3$, and it is still open for $n>3$. The notion of tame subgroup which is very much coordinate dependent must be replaced by the group $\mathop {\textrm{SAut}}{\mathbb {C}^n}$, generated by the flow maps (respectively the corresponding one-parameter subgroups) of locally nilpotent derivations, for short LNDs. The notion of a locally nilpotent derivation is coordinate-independent and makes sense on any affine algebraic variety, see Definition 2.3. The polynomial shears in Eq. (1) are examples of LNDs in $\mathbb {C}^n$. Understanding the importance of LNDs in affine algebraic geometry, a group of mathematicians introduced the notion of flexibility which proved extremely useful. The main result of their paper (Arzhantsev et al. 2013, Theorem 0.1) states the equivalence of the following three properties for an affine-algebraic manifold *X*:

1. *X* is flexible, i.e., the LNDs span the tangent space in each point.
2. The group $\mathop {\textrm{SAut}}(X)$ acts transitively.
3. The group $\mathop {\textrm{SAut}}(X)$ acts infinitely transitively, i.e., *m*-transitive for any $m \in \mathbb {N}$.

We denote the class of flexible manifolds by $\textrm{FLEX}$.

Concerning the above-mentioned question of Rosay–Rudin, the answer given by Andersén and Lempert is simply no, the group generated by shears and overshears in $\mathbb {C}^n$ is meagre in the holomorphic automorphism group $\mathop {\textrm{Aut}_\textrm{hol}}{\mathbb {C}^n}$ (even for $n=2$). However, the main result in their paper, the first version of the now so-called Andersén–Lempert Theorem, implies that the group generated by shears and overshears in $\mathbb {C}^n$ is dense in compact-open topology in the holomorphic automorphism group $\mathop {\textrm{Aut}_\textrm{hol}}{\mathbb {C}^n}$. The Andersén–Lempert Theorem, which in current form has been proved by Forstnerič and Rosay (1993a), led to a number of remarkable geometric constructions in $\mathbb {C}^n$. We refer the reader to the textbook of Forstnerič (2017) and to the overview articles of Kaliman and Kutzschebauch (2011) and Kutzschebauch (2014) for an account on this subject. As an interesting example, let us just name the existence of proper holomorphic embeddings of $\varphi :\mathbb {C}^k \hookrightarrow \mathbb {C}^n$ which are not straightenable, i.e., for no holomorphic automorphism $\alpha$ of $\mathbb {C}^n$ its image $\alpha (\varphi (\mathbb {C}^k))$ is equal to the first *k*-coordinate plane $\mathbb {C}^k \times \{0\}$. This in turn led to the negative solution of the holomorphic linearization problem. One can construct reductive subgroups of $\mathop {\textrm{Aut}_\textrm{hol}}{\mathbb {C}^n}$, which are not conjugated to a subgroup of linear transformations (for details see Derksen 1998).

The idea behind the Andersén–Lempert Theorem was generalized by Varolin to complex manifolds other than $\mathbb {C}^n$ (Varolin 2001). He introduced the notion of the density property, see Definition 2.6. This is a precise way of saying that the holomorphic automorphism group of a Stein manifold is large.

Methods from algebraic geometry turned out to be very fruitful in the search for manifolds with density property. Already Varolin had introduced the notion of algebraic density property for an affine algebraic manifold, which implies the density property. However, the algebraic density property is merely a tool for proving the density property, it does, for example, not imply flexibility since it is not using LNDs. For more details on flexibility see (Kutzschebauch 2014).

Some of the geometric constructions done in $\mathbb {C}^n$ with the help of the Andersén–Lempert Theorem could be generalized to Stein manifolds with the density property. However, there are constructions which still rely on the coordinates in $\mathbb {C}^n$. For example, the great embedding results of Riemann surfaces into $\mathbb {C}^2$ originating in the Ph.D. thesis of Wold which can be stated briefly as follows: If a bordered Riemann surface admits a non-proper holomorphic embedding into $\mathbb {C}^2$, then it also has a proper holomorphic embedding into $\mathbb {C}^2$ (Forstnerič and Wold 2009, Corollary 1.2). The geometric idea behind these results is to push the boundary of the bordered Riemann surface to infinity using a sequence of holomorphic automorphisms. To construct those automorphisms, a combination of the Andersén–Lempert Theorem with an explicitly given shear automorphism is used. The method of “precomposition with a shear” goes back to Buzzard and Forstnerič (1997), p. 161, and has been formalized in the notion of *nice projection property* in Kutzschebauch et al. (2009), Definition 2.1. All this research described above is part of a newly emerged area of elliptic holomorphic geometry, which also comprises Oka theory, the theory around the Oka–Grauert–Gromov homotopy principle. Stein manifolds with the density property are elliptic in the sense of Gromov, and, thus they are Oka manifolds. We refer to the textbook of Forstnerič (2017), Proposition 5.6.23 for details, see also Kutzschebauch (2014).

### The new notion

The aim of this paper is to introduce a new notion of largeness of the holomorphic automorphism group of an affine algebraic manifold. We call it *algebraic overshear density property*. It is stronger than the algebraic density property, in fact, it implies both the density property and the notion of flexibility introduced by Arzhantsev et al. (2013), p. 768. We are confident that this is the correct notion to generalize the geometric constructions known for $\mathbb {C}^n$ using both the “nice projection property” and Andersén–Lempert Theory to an affine-algebraic manifold *X*. Moreover, our notion fits in the modern point of view of affine algebraic geometry concentrating on LNDs and the group $\mathop {\textrm{SAut}}(X)$ generated by their flows. In addition to the powerful Andersén–Lempert Theorem we have the theory of locally nilpotent derivations to our disposal. For example, the existence of a quotient $\pi :X \rightarrow X//G_a$ which is quasi-affine according to Winkelmann (2003), and is a geometric quotient when restricted to an appropriate Zariski open subset (Vinberg and Popov 1989, Theorem 4.4) will be used in Sect. 5. This can be used to replace the use of shears in the “precomposition with a shear” trick.

The paper grew out of a discussion at the conference *Frontiers in Elliptic Holomorphic Geometry* held in Jevnaker which brought together researchers from affine algebraic geometry and elliptic holomorphic geometry. The idea to introduce a new notion closing the gap between different versions of flexibility in the two areas is due to Finnur Lárusson to whom we express our sincere gratitude. We also thank him for the careful reading of a first manuscript and proposing Problem 6.1.

The paper is organized as follows. In Sect. 2, we recall the definitions and define our new property. In the subsequent section we give some geometric consequences of the algebraic overshear density property and a criterion for it. In Sect. 4, we go through the list of affine algebraic manifolds known to have the algebraic density property, and explain which of them also have the algebraic overshear density property. his shows that our new property is strictly stronger than the algebraic density property. Moreover, we show that any algebraic vector bundle over an affine flexible base has the algebraic overshear density property. In Sect. 5, we prove that any smoothly bordered Riemann surface *R* with finitely many boundary components and non-empty boundary that is embedded in a complex affine surface *X* with the algebraic overshear density property, can be properly embedded into *X*. This generalizes the embedding result of Forstnerič and Wold (2009) for the case $X = \mathbb {C}^2$. In the last section, we propose some open problems. These mainly concern the geometric constructions which have been developed in $\mathbb {C}^n$ but not easily generalize to Stein manifolds with the density property.

## Definitions

### Definition 2.1

Let *X* be a complex manifold and let $\Theta$ be a $\mathbb {C}$-complete holomorphic vector field on *X*. Let $f \in \mathcal {O}(X)$ such that

1. $\Theta (f) = 0$, then $f \cdot \Theta$ is called a *shear of* $\Theta$ or a $\Theta$-*shear vector field*.
2. $\Theta ^2(f) = 0$, then $f \cdot \Theta$ is called an *overshear of* $\Theta$ or a $\Theta$-*overshear vector field*.

### Lemma 2.2

Let *X* be a complex manifold and let $\Theta$ be a $\mathbb {C}$-complete holomorphic vector field on *X*. Then the shears and overshears of $\Theta$ are also $\mathbb {C}$-complete holomorphic vector fields on *X*.

### Proof

See Varolin (1999), Proposition 3.2 for an abstract proof or see Andrist and Kutzschebauch (2018), Lemma 3.3 for an explicit formula of the flow maps. $\square$

### Definition 2.3

Let *X* be a complex affine variety. A *locally nilpotent derivation* (LND) on *X* is a $\mathbb {C}$-linear derivation $D :\mathbb {C}[X] \rightarrow \mathbb {C}[X]$ such that for each $f \in \mathbb {C}[X]$ there exists a number $n \in \mathbb {N}$ such that $D^n(f) = 0$.

We denote set of the *algebraic shear vector fields* of *X* by $\mathop {\textrm{LND}}(X)$. These are precisely the LNDs on *X*.

### Definition 2.4

Let *X* be a smooth complex affine variety (we call such a variety below a complex affine manifold).

1. The set of *holomorphic shear vector fields* of *X* consist of all holomorphic vector fields that are holomorphic shears of vector fields in $\mathop {\textrm{LND}}(X)$.
2. The set of *algebraic overshear vector fields* of *X* consist of all algebraic vector fields that are algebraic overshears of vector fields in $\mathop {\textrm{LND}}(X)$.
3. The set of *holomorphic overshear vector fields* of *X* consist of all holomorphic vector fields that are holomorphic overshears of vector fields in $\mathop {\textrm{LND}}(X)$.

### Definition 2.5

Let *X* be a complex affine manifold. We say that *X* enjoys the *algebraic overshear density property* if the Lie algebra generated by the algebraic overshear vector fields coincides with the Lie algebra of all algebraic vector fields. We denote the class of these manifolds by $\textrm{AOSD}$.

As a comparison, we give also the original definition of the (algebraic) density property introduced by Varolin (2001), Sect. 3.

### Definition 2.6

1. Let *X* be a complex affine manifold. We say that *X* enjoys the *algebraic density property* if the Lie algebra generated by the complete algebraic vector fields coincides with the Lie algebra of all algebraic vector fields.
2. Let *X* be a complex manifold. We say that *X* enjoys the *density property* if the Lie algebra generated by the complete holomorphic vector fields is dense (w.r.t. the topology of locally uniform convergence) in the Lie algebra of all holomorphic vector fields.

## Geometric consequences and a criterion

### Proposition 3.1

Let *X* be a complex affine manifold. If *X* has the algebraic overshear density property, then it is flexible.

### Proof

Let $x_0 \in X$ be a point. We will now find finitely many LNDs on *X* that span the tangent space in $x_0$.

Since *X* is affine, we can span the tangent space in $x_0$ by finitely many algebraic vector fields $\Theta _1, \dots , \Theta _n$ where $n = \dim X$.

The algebraic overshear density property implies that for each $j = 1, \dots , n$ there exist finitely many LNDs $\Theta _{j,1}, \dots , \Theta _{j, m(j)}$ and regular functions $f_{j,1} \in \ker \Theta _{j,1}^2, \dots , f_{j,m(j)} \in \Theta _{j, m(j)}^2$ such that each $\Theta _j$ is a Lie combination of $f_{j,k} \cdot \Theta _{j,k}$ where $k = 1, \dots , m(j)$.

We make the following observation: Let $f \cdot \Theta$ resp. $\widetilde{f} \cdot \widetilde{\Theta }$ be overshears of LNDs $\Theta$ resp. $\widetilde{\Theta }$. Then we obtain for their Lie bracket:

$$\begin{aligned} \left[ f \cdot \Theta , \widetilde{f} \cdot \widetilde{\Theta } \right] = f \Theta (\widetilde{f}) \cdot \widetilde{\Theta } - \widetilde{f} \widetilde{\Theta }(f) \cdot \Theta + f \widetilde{f} \left[ \Theta , \widetilde{\Theta } \right] \end{aligned}$$

Hence, we see that each vector $\Theta _j | x_0$ is a complex linear combination of the LNDs $\Theta _{j,k}$ and their Lie brackets, evaluated in $x_0$. Moreover, a Lie bracket of two LNDs $\Theta$ and $\widetilde{\Theta }$ can be expressed using the flow map $\varphi _t$ of $\Theta$:

$$\begin{aligned} \left[ \Theta , \widetilde{\Theta } \right] _{x} = \lim _{t \rightarrow 0} \frac{ \mathop {d \varphi _{-t}} \widetilde{\Theta }_{\textstyle \varphi _t(x)} - \widetilde{\Theta }_{\textstyle x}}{t} \end{aligned}$$

Since $\Theta$ is an LND, its flow $\varphi _t$ is algebraic, and we can approximate $\left[ \Theta , \widetilde{\Theta } \right]$ arbitrarily well by the sum of two LNDs. Applying these arguments inductively, we see that each vector $\Theta _j | x_0$ can be approximated arbitrarily well by a complex linear combination of LNDs. Since spanning the tangent space is an open condition, we conclude that finitely many LNDs span the tangent spaces in an open neighborhood of $x_0$.

The set where these LNDs do not span the tangent spaces, must be an algebraic subvariety. Hence, we obtain the desired conclusion by a standard argument. $\square$

We will need the following notion of a *compatible pair* which was introduced by Kaliman and Kutzschebauch (2008a), Definition 2.5:

### Definition 3.2

Let *X* be an affine algebraic manifold *X*. A *compatible pair of LNDs* are $\Theta , \, \Xi \in \mathop {\textrm{LND}}(X)$ such that

1. $$\exists \, h \in (\ker \Theta ^2 {\setminus } \ker \Theta ) \cap \ker \Xi $$
2. $\mathop {\textrm{span}}_\mathbb {C}\{ \ker \Theta \cdot \ker \Xi \}$ contains a non-trivial $\mathcal {O}_{\text {alg}}(X)$-ideal

### Proposition 3.3

Let *X* be an affine algebraic manifold that is flexible and a admits a compatible pair of LNDs. Then *X* has the algebraic overshear density property.

### Proof

The proof follows the lines of proof of Theorems 1 and 2 from Kaliman and Kutzschebauch (2008a). The compatible pair creates a submodule of the algebraic vector fields which is contained in the Lie algebra generated by algebraic overshear vector fields. Then transitivity of $\mathop {\textrm{SAut}}(X)$ follows from flexibility, and the transitivity on a tangent space is also an easy consequence of flexibility. In fact, in Arzhantsev et al. (2013), Theorem 4.14 and Remark 4.16 even more is proven, namely that any element in $\textrm{SL}(T_p X)$ is contained in the isotropy group of $\mathop {\textrm{SAut}}(X)$ at a point $p \in X$. Since the pull-back of an LND by an algebraic automorphism is again an LND, the above reasoning shows that the Lie algebra generated by algebraic overshear vector fields is equal to the Lie algebra of all algebraic vector fields on *X*. $\square$

### Theorem 3.4

Let *X* be a complex affine manifold with the algebraic overshear density property. Then the group generated by flows of algebraic overshear vector fields is dense in the identity component $\mathop {\textrm{Aut}_\textrm{1}}(X)$ in compact-open topology.

### Proof

This is a direct application of the Andersén–Lempert Theorem, which can be found implicitly in Andersén and Lempert (1992), Forstnerič and Rosay (1993a, 1993b), and Varolin (2001). For $\mathbb {C}^n, n \ge 2,$ the best explicit reference is Forstnerič (2017), Theorem 4.9.2 with $\Omega = \mathbb {C}^n$. For the general case, the analogous theorem can be found explicitly in Kaliman and Kutzschebauch (2011), Theorem 2 where one chooses $\Omega = X$ and any $\Phi _1 \in \mathop {\textrm{Aut}_\textrm{1}}(X)$. Only the flows of those complete vector fields are used for the approximation that were needed to establish the density property. This is implicit in the proof of Kaliman and Kutzschebauch (2011), Theorem 2. $\square$

### Theorem 3.5

Let *X* be a complex affine surface with the algebraic overshear density property and let *Y* be a complex manifold. If the holomorphic endomorphisms of *X* and the holomorphic endormophisms of *Y* are isomorphic as semi-groups, then *X* and *Y* are biholomorphic or anti-biholomorphic.

### Proof

A general orbit of the $\mathbb {C}^+$-action generated by an LND gives an algebraic embedding of $\mathbb {C}\hookrightarrow X$ which is automatically proper. The theorem then follows from a theorem of Andrist (2011), Theorem 3.3. $\square$

### Remark 3.6

For a manifold *X* with the density property there exists a proper holomorphic immersion $\mathbb {C}\rightarrow X$. If in addition $\dim X \ge 3$, then there exists a proper holomorphic embedding $\mathbb {C}\rightarrow X$ (see Andrist and Wold 2014, Theorem 5.2) and the above theorem follows. If $\dim X = 2$, we do not know in general, whether a proper holomorphic embedding $\mathbb {C}\rightarrow X$ exists.

## (Algebraic) density versus Algebraic overshear density: all known examples

We will go through the list of known examples of affine-algebraic manifolds with the density property, and indicate for each one of them whether or not they have the algebraic density property resp. the algebraic overshear density property. As a general feature one can see that the gap between algebraic density property and algebraic overshear density property as rather narrow, but existent.

1. A homogeneous space $X=G/H$ where *G* is a linear algebraic group and *H* is a closed algebraic subgroup such that *X* is affine and whose connected components are different from $\mathbb {C}$ and from $(\mathbb {C}^*)^n, n\ge 1,$ has algebraic density property.
  It is known that if *H* is reductive, then the space $X=G/H$ is always affine. However, there is no known group-theoretic criterion that would characterize when *G*/*H* is affine. The above result has a long history, it includes all examples known from the work of Varolin (2001), Toth and Varolin (2000), Kaliman and Kutzschebauch (2008a), and Donzelli et al. (2010). The final result has been obtained by Kaliman and Kutzschebauch (2017). The one-dimensional manifolds $\mathbb {C}$ and $\mathbb {C}^*$ do not have the density property, however the following problem is well known and seems notoriously difficult:
  **Open Problem:** Does $(\mathbb {C}^*)^n, n\ge 2,$ have the density property?
  It is conjectured that the answer is no, more precisely one expects that all holomorphic automorphisms of $(\mathbb {C}^*)^n, n\ge 2$ preserve the form $\wedge _{i=1}^n \frac{ \textrm{d} z_i}{ z_i}$ up to sign.

We are able to characterize those homogeneous spaces *X* from above which have the algebraic overshear density property.

### Theorem 4.1

Let $X = G/H$ be an affine homogeneous space of a connected linear algebraic group $G < \textrm{GL}_n(\mathbb {C})$ by a closed algebraic subgroup *H*. Then *X* has the algebraic overshear density property if and only if all algebraic morphisms $X \rightarrow \mathbb {C}^*$ are trivial.

### Proof

Since the algebraic maps from $\mathbb {C}$ to $\mathbb {C}^\star$ are constant, the flow curves of an LND have to be tangent to the fibers of any algebraic morphism $X \rightarrow \mathbb {C}^*$. This proves the necessity part. To see the sufficiency of the condition, we follow the proof of the algebraic density property for *X* of Kaliman and Kutzschebauch (2017), Theorem 11.7. That proof needs only LNDs and their algebraic overshears if (and only if) the torus $T_1$ introduced in Kaliman and Kutzschebauch (2017), Notation 4.10, p. 1319 is trivial, which in turn follows from the triviality of all algebraic morphisms $X \rightarrow \mathbb {C}^*$, and by Kaliman and Kutzschebauch (2017), Proposition 5.2. *X* is isomorphic to $Z \times T_1$ where *Z* is an affine flexible variety by Kaliman and Kutzschebauch (2017), Lemma 4.11. Thus, by Proposition 3.3 it suffices to establish existence of a compatible pair of LNDs on *Z*. There are two cases, and in the first one such existence follows from Kaliman and Kutzschebauch (2017), Proposition 7.1. In the second one, there is a construction of compatible pairs in the proof of Kaliman and Kutzschebauch (2017), Corollary 8.2 where the assumptions of the latter corollary are checked for *Z* in the proof of Kaliman and Kutzschebauch (2017), Theorem 10.6. $\square$

### Example 4.2

(for Theorem 4.1)

- The motivating example is of course $\mathbb {C}^n$, $n \ge 2$, where it is sufficient to take algebraic overshears of $\frac{\partial }{\partial z_1}, \dots , \frac{\partial }{\partial z_n}$ to establish the algebraic overshear density property as it was shown by Andersén and Lempert (1992).
- $\textrm{GL}_n(\mathbb {C}), n \ge 2,$ has the algebraic density property, but not the algebraic overshear density property, since $\det :\textrm{GL}_n(\mathbb {C}) \rightarrow \mathbb {C}^*$ is non-trivial.
- $\textrm{SL}_n(\mathbb {C}), n \ge 2,$ has the algebraic overshear density property.

We continue our list of all known examples for the algebraic overshear density property.

(2) The manifolds *X* given as a submanifold in $\mathbb {C}^{n+2}$ with coordinates $u\in \mathbb {C}$, $v\in \mathbb {C}$, $z\in \mathbb {C}^n$ by the equation $uv = p(z)$, where the zero fiber of the polynomial $p \in \mathbb {C}[\mathbb {C}^n]$ is smooth (otherwise *X* is not smooth) have the algebraic density property and, thus, the density property (Kaliman and Kutzschebauch 2008b). We claim that all these examples have the algebraic overshear density property:
  - For $n=1$ this is the main result of Kutzschebauch and Lind (2011), Sect. 3. Note that for $n=1$ this is also an example of a complex manifold with density property that does not admit compatible pairs (see Definition 3.2) which follows from Proposition 2.9 in Kaliman and Kutzschebauch (2016).
  - For $n \ge 2$ it is an easy exercise to show that the group $\mathop {\textrm{SAut}}(X)$ acts transitively on *X* and that the pairs of LNDs $(\theta _i, \theta _j), i \ne j$ form compatible pairs where $\theta _i:= \frac{\partial p}{ \partial z_i} \frac{\partial }{\partial u} - u \frac{\partial }{\partial z_i}$. The result now follows from Proposition 3.3.
(3) Before formulating the next result, recall that *Gizatullin surfaces* are by definition the normal affine surfaces on which the algebraic automorphism group acts with an open orbit whose complement is a finite set of points. By the classical result of Gizatullin, they can be characterized by admitting a completion with a simple normal crossing chain of rational curves at infinity. Every Gizatullin surface admits a $\mathbb {C}$-fibration with at most one singular fibre which is however not always reduced.
  Smooth Gizatullin surfaces which admit such a $\mathbb {C}$-fibration that the singular fibre is reduced (sometimes called *generalized Danielewski surfaces*) have the density property (Andrist 2018). We do not know except for the case of Danielewski surfaces whether they have the algebraic overshear density property, since in the proof of the density property one uses LNDs pulled back by certain holomorphic (non-algebraic) automorphisms arising as flow maps of algebraic vector fields. These pullbacks are not necessarily algebraic vector fields.
(4) Certain algebraic hypersurfaces in $\mathbb {C}^{n+3}$:
  $$\begin{aligned} \{ (x, y, z_0, z_1, \dots , z_n) \in \mathbb {C}\times \mathbb {C}\times \mathbb {C}^{n+1} :\, x^2 y = a(z) + x \cdot b(z) \} \end{aligned}$$
  where $\deg _{z_0} a \le 2$, $\deg _{z_0} b \le 1$ and not both degrees are zero. This class includes the Koras–Russell cubic threefold and has the density property according to Leuenberger (2016).
  The Koras–Russell cubic $\{ x + x^2 y + u^2 + v^3 = 0 \} \subset \mathbb {C}^4_{x,y,u,v}$ which is famous for being diffeomorphic to $\mathbb {R}^6$, but being not algebraically isomorphic to $\mathbb {C}^3$ cannot be flexible and, hence, does not have the algebraic overshear density property, since all LNDs necessarily fix the *x*-coordinate axis, see Makar-Limanov (1996). We do not know whether those examples of Leuenberger have algebraic density property by the same reason as in the class of examples (3) above.
(5) The Calogero–Moser spaces $\mathcal {C}_n$ have the algebraic overshear density property. An inspection of the proof in Andrist (2021) shows that only overshears are needed: The density property follows from the existence of a non-degenerate algebraic $\textrm{SL}_2(\mathbb {C})$ action and from flexibility. Hence, all the involved vector fields are overshears of LNDs.

Recently, Ugolini and Winkelmann proved that if *X* is a complex affine manifold that is flexible, and if $E \rightarrow X$ is an algebraic vector bundle over *X*, then *E* is flexible as well (Ugolini and Winkelmann 2022, Theorem 1.6). Moreover, they proved that if *X* is a complex affine manifold with the density property, and if *E* is algebraic and flexible, then *E* has the density property (Ugolini and Winkelmann 2022, Corollary 1.5). Thus, if *X* has the algebraic overshear density property, then it follows from their results that the total space *E* of an algebraic vector bundle $E \rightarrow X$ has the density property. However, their proof requires as an essential ingredient the Euler vector field which is a $\mathbb {C}^*$-action and not an LND. This leads naturally to the question whether the total space *E* admits the overshear density property. Indeed, this is true by our next result.

### Theorem 4.3

Let *X* be a flexible complex affine manifold. Let $E \rightarrow X$ be an algebraic vector bundle over *X*. Then *E* has the algebraic overshear density property.

Before we start with the proof let us remind some general construction for an LND $\Theta$ acting on an affine-algebraic variety *X*.

### Remark 4.4

We quote the following from Arzhantsev et al. (2013), Remarks 2.7: According to Vinberg and Popov (1989), Theorem 3.3 the field of rational invariants is the quotient field of the ring $\mathbb {C}[X]^\Theta$ of regular invariants for an affine-algebraic variety *X* for an LND $\Theta$. Hence by a corollary of the Rosenlicht theorem on rational invariants (see Vinberg and Popov 1989, Proposition 3.4) the regular invariants $\mathbb {C}[X]^\Theta$ separate orbits on an $\Theta$-invariant open dense subset $U(\Theta )$ of *X*. Shrinking $U(\Theta )$ if necessary we can achieve that $U(\Theta )$ has a geometric quotient $U(\Theta )/\Theta$, which admits a locally closed embedding into some $\mathbb {C}^N$ by regular invariants in $\mathbb {C}[X]^\Theta$ (see also Vinberg and Popov 1989, Theorem 4.4).

### Proof of Theorem 4.3

We choose a non-trivial LND $\Theta$ on *X* which exists due to flexibility. We now apply the Remark 4.4 above to the $(\mathbb {C}, +)$-action induced by $\Theta$ and obtain an $\Theta$-invariant open dense subset $U(\Theta )$ of *X*. Also by the same Rosenlicht theorem (see e.g. Springer 1998, Proposition 14.2.2) we have an isomorphism $\Phi :\mathbb {C}_z \times Y \rightarrow U(\Theta )$ where *Y* is an affine-algebraic variety *Y*, and $\Phi ^*(\Theta ) = \frac{\partial }{\partial z}$.

Moreover, the algebraic vector bundle $\pi :E \rightarrow X$ is trivial over a non-empty Zariski-open subset $V \subset X$. In particular, the bundle is trivial over $U(\Theta ) \cap V$. On $\mathbb {C}\times Y$, we can extend the trivialization from $\Phi ^{-1}(U(\Theta ) \cap V)$ to $\mathbb {C}\times Y'$ for some non-empty Zariski-open subset $Y' \subset Y$ using the action of $\Phi ^*(\Theta )$ and the fact that an algebraic vector bundle is invariant under the flow of an LND of the base space (see e.g. Ugolini and Winkelmann 2022, Theorem 11.1).

We set $W:= \Phi (\mathbb {C}\times Y')$. Since $W \subset X$ is a $\Theta$-invariant Zariski-open subset of the affine variety $U(\Theta )$, there exists a non-zero regular function $f :X \rightarrow \mathbb {C}$ with $W \subset \{f \ne 0\}$ that is $\Theta$-invariant: In fact, we can find a non-zero function which is a polynomial in the finitely many regular invariants of $\mathbb {C}[X]^\Theta$ that embed $Y \cong U(\Theta )/\Theta$ as a locally closed subset into $\mathbb {C}^N$.

Let $r \ge 1$ be the rank of *E*. A dense affine subset of *E* is now given by $W \times \mathbb {C}^r \cong (\mathbb {C}_z \times Y') \times \mathbb {C}^r_w$. The subscripts indicate the variable names, i.e. $z \in \mathbb {C}_z \cong \mathbb {C}$ and $w = (w_1, \dots , w_r) \in \mathbb {C}^r_w \cong \mathbb {C}^r$. By $\hat{\Phi }$ we also denote the extension of the isomorphism $\Phi$ to the trivial bundle, $\hat{\Phi } :(\mathbb {C}_z \times Y') \times \mathbb {C}^r_w \rightarrow W \times \mathbb {C}^r$. Let $\Xi := \hat{\Phi }_*\frac{\partial }{\partial w_1}$. Then $\widetilde{\Theta }:= (f \circ \pi ) \cdot \hat{\Phi }_*\Phi ^*\Theta$ and $\widetilde{\Xi }:= (f \circ \pi ) \cdot \Xi$ are well defined algebraic vector fields on *E* and are in fact LNDs. On $(\mathbb {C}_z \times Y') \times \mathbb {C}^r_w$, the vector fields $\frac{\partial }{\partial z}$ and $\frac{\partial }{\partial w_1}$ form a compatible pair, since the span of the product of their kernels contains in fact all regular functions. Hence, $\widetilde{\Theta }$ and ${\widetilde{\Xi }}$ form a compatible pair on *E*, since the span of the product of their kernels contains the ideal generated by $f \circ \pi$.

Moreover, we have flexibility of the total space *E* by Ugolini and Winkelmann (2022), Theorem 11.4, and hence the result follows from Proposition 3.3. $\square$

## Embedding bordered Riemann surfaces

We generalize the following result from $\mathbb {C}^2$ to complex-affine surfaces with the algebraic overshear density property.

### Theorem 5.1

Let *X* be a complex affine surface with the algebraic overshear density property and let $R \subset X$ be an embedded, smoothly bordered Riemann surface with finitely many boundary components and non-empty boundary. Then there exists a proper holomorphic embedding of the Riemann surface *R* into *X*.

### Remark 5.2

The exact assumption in the Theorem is that $R^\circ$ is the interior of a closed domain with boundary of class $\mathcal {C}^r$, $r \ge 2$, in a compact (not necessarily connected) Riemann surface ${\hat{R}}$. We may assume that the boundary consists of real analytic Jordan curves, and *R* has no connected component without boundary, see Stout (1965), Theorem 8.1. Note that any conformal diffeomorphism of *R* onto such a domain is in $\mathcal {C}^1(\hat{R})$; see Alarcón et al. (2021), Theorem 1.10.10. By *embeddded*, we mean that there exists an injective and immersive $\mathcal {C}^1$-map $f :R \rightarrow X$ that is holomorphic in the interior $R^\circ$ of *R*. By an application of the Mergelyan theorem as in the proof of Corollary 1.2 in Forstnerič and Wold (2009) we may assume that *f* is, in fact, holomorphic in an open neighborhood *U* of *R* in $\hat{R}$. The corresponding form of the manifold-valued Mergelyan Theorem can be found in Fornæss et al. (2020), Corollary 8 on p. 178.

### Remark 5.3

A complex-affine surface *X* with the algebraic overshear density property has trivial Makar-Limanov invariant, since it is flexible (Arzhantsev et al. 2013, Proposition 5.1). For a normal complex-affine surface non-isomorphic to $\mathbb {C}\times \mathbb {C}^*$ and $\mathbb {C}^*\times \mathbb {C}^*$, this is one of the equivalent conditions implying that *X* is a Gizatullin surface (see Bandman and Makar-Limanov 2001 in the smooth case, and Dubouloz 2004 in the normal case). Together, this implies that our *X* above is a homogeneous Gizatullin surface. We will however not make use of this fact in the following proof. We do not know whether the result of Theorem 5.1 extends to all homogeneous Gizatullin surfaces. Our proof uses the density property which is not established for all homogeneous Gizatullin surfaces. The first author proved that Gizatullin surfaces with reduced degenerate fibre have the density property (Andrist 2018) which does not cover all homogeneous Gizatullin surfaces.

Among the ingredients, we will need Zariski’s finiteness theorem:

### Theorem 5.4

(Zariski 1954, cited after Freudenburg 2017, Sect. 6.3). For a field *k*, let *A* be an affine normal *k*-domain, and let *K* be a subfield of $\textrm{frac}(A)$ containing *k*. If $\mathrm {tr.deg.}_k K \le 2$, then $K \cap A$ is finitely generated over *k*.

### Corollary 5.5

Let *X* be a complex-affine surface, and let $\Theta \in \mathop {\textrm{LND}}{X}$. Then the GIT quotient $X /\!/ \mathbb {C}^+_\Theta$ is affine.

Any non-trivial LND $\Theta$ admits a *local slice*, i.e., in our context a polynomial function $f :X \rightarrow \mathbb {C}$, such that $\Theta (f) \ne 0$ but $\Theta ^2(f) = 0$. If the GIT quotient $X /\!/ \mathbb {C}^+_\Theta$ is affine, then there exists a Zariski-open subset *U* in the quotient s.t. the corresponding fibration over *U* is trivial and each such fibre is isomorphic to $\mathbb {C}$, see Freudenburg (2017), Principle 11, p. 27 and the comment (Freudenburg 2017, p. 34).

The proof of the main theorem of this section uses the idea of a “pre-composition with a shear” from the original proof of Forstnerič and Wold in the case of $X = \mathbb {C}^2$ (Forstnerič and Wold 2009). It has been generalized to $X = \mathbb {C}\times \mathbb {C}^*$ by Lárusson (2014) and to $X = (\mathbb {C}^*)^2$ by Ritter (2018). We give a short outline of their proof and indicate the necessary adjustments to our situation.

### Proof

According to Proposition 3.1, we can choose two LNDs $\Theta _1$ and $\Theta _2$ on *X* that are not proportional. Denote by $\pi _k :X \rightarrow X /\!/ \mathbb {C}^+_{\Theta _k}$ the projection to the GIT quotient of $\Theta _k$ for $k=1,2$. Note the following facts:

1. $X /\!/ \mathbb {C}^+$ is a smooth affine curve, since *X* is a smooth surface, and, thus, the quotient is normal which excludes singularities. In addition, the existence of a second non-proportional locally nilpotent derivation gives a non-constant map of $\mathbb {C}$ (any generic orbit of this second LND) into $X /\!/ \mathbb {C}^+$ and, thus, $X /\!/ \mathbb {C}^+$ is isomorphic to $\mathbb {C}$.
2. The general fibres of $\pi _k$ are isomorphic to $\mathbb {C}$ with only finitely many special fibres.

In the first step of the proof, we expose points w.r.t. $\pi _2$. We indicate the necessary adjustments to the method introduced by Forstnerič and Wold (2009), Sect. 4.

### Definition 5.6

A point $p \in \partial R$ is called *exposed* w.r.t. $\pi _k \; (k = 1, 2)$ if

$$\begin{aligned} \pi _k^{-1} (\pi _k (p)) \cap \overline{R} = \{p\} \end{aligned}$$

Moreover, $F_p:=\pi _k^{-1} (\pi _k (p))$ and the boundary $\partial R$ intersect transversely, in fact $T_p F_p \cap T_p \partial R =\{0\}$.

Following the procedure in Forstnerič and Wold (2009), Sect. 4 we change the embedding of *R* into *X* so that in each of the finitely many boundary curves $(\partial R)_i$ there exists an exposed point $p_i$ and such that each of the exposed points lies in a general fibre of $\pi _2$. The proof goes exactly as the proof of Theorem 4.2 in Forstnerič and Wold (2009). The conclusion that $p_i$ is in a general fibre can be easily achieved by adding this assumption to choice of the points $p_i$ (same notation in the proof of Theorem 4.2) before choosing the arcs $\lambda _i$ in that proof (see also Fig. 2 in Forstnerič and Wold 2009). The Mergelyan Theorem for maps to $\mathbb {C}^2$ (functions) should be replaced by the (Oka) manifold-valued Mergelyan Theorem proved by Forstnerič and Wold (2019) which can be found in Fornæss et al. (2020), Corollary 8. By the definition of an exposed point we then have $\pi _2(p_i) \ne \pi _2(p_j)$ for $p_i \ne p_j$.

In the second step, we follow the procedure in Forstnerič and Wold (2009), Sect. 5 which in turn relies on Wold (2006), Sect. 4, in particular Lemma 1 therein. We consider $\pi _1$ restricted to $\pi _2^{-1}\{ \textrm{q} \}$ for a point $q \in X /\!/ \mathbb {C}^+_{\Theta _2} \cong \mathbb {C}$ where in fact we will choose $q = \pi _2(p_i)$. Since this fibre is isomorphic to $\mathbb {C}$ as well, we can find algebraic coordinates and write $\pi _1 | \pi _2^{-1}\{ \textrm{q} \}$ as a polynomial map $\mathbb {C}\rightarrow \mathbb {C}$. Choosing a sufficiently small Euclidean neighborhood $D \subset \mathbb {C}$ around *q* such that all fibers above points in *D* are general, we can write

$$\begin{aligned} \pi _1(z,w) = a_n(w) \cdot z^n + a_{n-1}(w) \cdot z^{n-1} + \dots + a_1(w) \cdot z + a_0(w) \end{aligned}$$

where $w \in D$ and $z \in \mathbb {C}$ is in the fibre above *w* w.r.t. $\pi _2(z,w) = w$. The coefficients $a_i \in \mathcal {O}(D)$ are holomorphic functions. Note that this map can’t be of degree 0 in *z* since the LNDs $\Theta _1$, $\Theta _2$ are not proportional.

Let $\varphi _2(x,t)$ with time $t \in \mathbb {C}$ and $x \in X$ be the (complete) flow of $\Theta _2$.

Consider the map

$$\begin{aligned} x \mapsto \Phi (x):= \varphi _2 \left( x, \sum _{i} \frac{\alpha _i}{\pi _2(x) - \pi _2(p_i)} \right) \end{aligned}$$

with $\alpha _i \in \mathbb {C}{\setminus } \{0\}$. When restricted to *R*, this sends precisely the exposed points to infinity. The choices of the finitely many constants $\alpha _i$ have to be made such that the $\pi _1$-projections of the paths given by “the opened boundary components” $u_i :\mathbb {R}\rightarrow \Phi ((\partial R)_i)$ are pairwise disjoint outside a compact. Note that these projections lie in the quotient isomorphic to $\mathbb {C}$. Hence, we can proceed with the choice of constants $\alpha _i$ as in the original proof for $\mathbb {C}^2$ where the projection was onto a coordinate axis; we just need to notice the following:

Without loss of generality, we may assume $q = 0$ in suitable coordinates for *D*. We locally (around the exposed point) parametrize the curve $(\partial R)_i$ through the exposed point by $\gamma _i = (\gamma _i^1,\gamma _i^2)$ and assume $\gamma _i^1(0) = q = 0$. Let $\gamma _i^1(t) = a_i^1 t + a_i^2 t^2 + \dots$ be the Taylor expansion in *t* centered at 0. For small enough *D* we can assume that

$$\begin{aligned} \sum _{i} \frac{\alpha _i}{\pi _2(x) - \pi _2(p_i)} = \frac{\alpha _{i}}{w} + O(1) \end{aligned}$$

We then obtain for $t \rightarrow 0$ that

$$\begin{aligned} \pi _1(\Phi (\gamma _i(t))) = a_n(\gamma _i^2(t)) \cdot (\gamma _i^1(t))^n + \dots = \left( \frac{\alpha _i}{a_i^1}\right) ^n \frac{1}{t^n} + \dots \end{aligned}$$

Our calculations show that the assumptions of Wold (2006), Lemma 1 are satisfied; In particular, for *t* close to zero, we obtain that each projection $\pi _1(\Phi (\gamma _i(t)))$ is $\mathcal {C}^1$-close to the union of two different straight rays $L_1$, $L_2$ going to infinity in opposite directions (one ray for $t\rightarrow 0^+$ and another ray for $t\rightarrow 0^-$). As said above by choosing the coefficients $\alpha _i$ accordingly, we achieve that all those rays for all the exposed points are different. Thus, outside a big disc $\Delta _R \subset X /\!/ \mathbb {C}^+_{\Theta _1} \cong \mathbb {C}$, the images $\pi _1(\Phi (\gamma _i(t)))$ of the opened boundary curves together with $\Delta _R$ form a “Mergelyan set”, i.e., the complement has no bounded components. We are not using Wold (2006), Lemma 1 as it stands, only the strategy of its proof. The necessary adjustments for our case are indicated in the proof of Lemma 5.7 below.

In the third step, we consider now the “opened” boundary curves $u_i :\mathbb {R}\rightarrow X$. Our aim is to push them to infinity by applying inductively a sequence of automorphisms of *X* while keeping the interior of *R* in the domain of convergence of this sequence.

Since *X* is a Stein manifold, we can exhaust it by $\mathcal {O}(X)$-convex compacts $\{X_m\}_{m \in \mathbb {N}}$ such that $X_m \subset X_{m+1}^\circ$ and $\cup _{m \in \mathbb {N}} X_m = X$. Similarly, we exhaust *R* by compacts $R_m$.

We will apply the following lemma inductively:

### Lemma 5.7

There exists a holomorphic automorphism $\alpha :X \rightarrow X$ such that for any given $\mathcal {O}(X)$-convex compacts $K \subset L \subset X$ and any $\varepsilon > 0$, we obtain that

1. $\Vert \alpha - \mathop {\textrm{id}}\Vert _K < \varepsilon$ (w.r.t. any fixed embedding of *X* into some $\mathbb {C}^N$)
2. $\alpha \circ u_i(\mathbb {R}) \cap L = \emptyset$ for every opened boundary curve $u_i :\mathbb {R}\rightarrow X$.

### Proof

We obtain $\alpha$ in the form $\alpha = f \circ s$ where *s* is a shear map. This is the so called “precomposition with a shear” trick invented by Buzzard and Forstnerič (1997). First, the automorphism *f* is constructed using Andersén–Lempert theory as in Wold (2006), Lemma 1 to expell a big but still compact part of the opened boundary curve from the compact *L* in *X*. Note that the proof does not make use of the linear structure of $\mathbb {C}^2$. Also, their application of Stolzenberg’s result (Stolzenberg 1966) on the holomorphic convexity of the union of a holomorphically convex compact and finitely many smooth real arcs attached to it, is justified in our situation, since it holds in any Stein manifold instead of $\mathbb {C}^2$, see for example the points (1) and (2) in the proof of Forstnerič and Wold (2009), Theorem 5.1.

In the second step, the precomposing shear map *s* is constructed as the time-1 map of a holomorphic shear $h \Theta _1$: Here, the holomorphic function *h* which is in the kernel of the LND $\Theta _1$, is obtained by using Mergelyan’s theorem. It is used to ensure that no parts from “far away” in the opened boundary curves can come back to the compact set $L\subset X$ (recall that Andersén–Lempert theory gives only approximation on compacts). Indeed, we verified in the second step above that the assumptions of Wold (2006), Lemma 1 are satisfied, which allows the use of Mergelyan’s theorem. Note that the corresponding GIT quotient is isomorphic to $\mathbb {C}$ and the set $\mathbb {C}{\setminus } (\Delta _R \cup \pi _1 (u_i))$ has no bounded components. Moreover, we choose *R* big enough so that the finitely many image points of the exceptional fibers $E_1, \dots , E_r$ of $\pi _1$ are contained in $\Delta _R$. Hence, we can apply Mergelyan’s theorem in one dimension. $\square$

The final embedding of *R* is now obtained by applying Lemma 5.7 for each $K = X_m \cup (\alpha _{m-1} \circ \dots \circ \alpha _1)(R_{j(m)})$, $j(m)\ge m$ and $L = X_{m'}$, inductively in $m \in \mathbb {N}$ as in Forstnerič and Wold (2009), Theorem 5.1, p. 111. Here, $j(m) \in \mathbb {N}$ is chosen big enough such that *K* is polynomially convex, see point (2) in the proof of Forstnerič and Wold (2009), Theorem 5.1, p. 111, and where $m' \ge m$ is chosen such that $X_{m'} \supset K$ in each step.

For each $m \in \mathbb {N}$ we obtain an automorphism $\alpha _m :X \rightarrow X$ such that $\Vert \alpha _m - \mathop {\textrm{id}}\Vert _{X_m \cup (\alpha _{m-1} \circ \dots \circ \alpha _1)(R_{m(j)})} < 2^{-m}$ as well as $\alpha _m \circ (\alpha _{m-1} \circ \dots \circ \alpha _1 \circ u_i(\mathbb {R})) \cap X_{m'} = \emptyset$ for every opened boundary curve $u_i$.

Then the limit $g:= \lim _{m \rightarrow \infty } \alpha _m \circ \alpha _{m-1} \circ \dots \circ \alpha _1$ converges uniformly on compacts of a Fatou–Bieberbach domain $\Omega := \bigcup _{m \in \mathbb {N}} (\alpha _m \circ \alpha _{m-1} \circ \dots \circ \alpha _1)^{-1}(X_m)$ and defines a biholomorphic map $g :\Omega \rightarrow X$ (see Prop. 4.1.1. from Forstnerič (2017). This domain does not contain the opened boundary curves since they are pushed out of the compact $X_{m'}$ in step *m* (thus going to infinity). However, *R* is inside the domain of convergence, since the maps $\alpha _m$ are closer and closer to identity on the exhausting compact subsets $R_{j(m)}\supset R_m$. Thus, the restriction $g\vert _R :R \rightarrow X$ is a proper holomorphic embedding of the open Riemann surface *R* into *X*. $\square$

## Open problems

Recall that we denote the class of flexible manifolds by $\textrm{FLEX}$ and the class of manifolds with the algebraic overshear density property by $\textrm{AOSD}$. For the following problem, let us moreover denote the class of manifolds with the algebraic density property by $\textrm{AD}$. We know that $\textrm{AD} {\setminus } \textrm{FLEX} \ne \emptyset$, for e.g. $\mathbb {C}\times \mathbb {C}^*$ is in this set, see Arzhantsev et al. (2013) for a larger list of examples. By Proposition 3.1 we know that $\textrm{AD} \cap \textrm{FLEX} \subseteq \textrm{AOSD}$. However, we do not know whether this inclusion is strict. This motivates the following problem.

### Problem 6.1

1. Is $\textrm{FLEX} {\setminus } \textrm{AD} \ne \emptyset$ among affine-algebraic manifolds?
2. Is $$(\textrm{AD} \cap \textrm{FLEX}) {\setminus } \textrm{AOSD} \ne \emptyset $$?

### Definition 6.2

Let *X* be a complex manifold and $\tau \in \mathop {\textrm{Aut}}{X}$. We call $\tau$ a *generalized translation* if for any $\mathcal {O}(X)$-convex compact $K \subsetneq X$ there exists an $m \in \mathbb {N}$ such that for the iterate $\tau ^m$ it holds that

1. $$\tau ^m(K) \cap K = \emptyset $$ and
2. $$\tau ^m(K) \cup K$$ is $$\mathcal {O}(X)$$-convex.

### Problem 6.3

Let *X* be a complex-affine manifold with the algebraic overshear density property. Does there exist a generalized translation (Andrist and Wold 2015, Definition 1.4) on *X*? This would imply that there exist two automorphisms that generate a dense subset of the identity component of the automorphism group $\mathop {\textrm{Aut}_\textrm{1}}(X)$.

### Problem 6.4

Let *X* be a complex-affine manifold with the algebraic overshear density property and with $\dim X \ge 3$. Does there exist a compatible pair of LNDs on *X*?

The result of Andersén and Lempert, answering the question of Rosay and Rudin mentioned in the introduction, says that the group generated by overshears is meagre in $\mathop {\textrm{Aut}_\textrm{hol}}(\mathbb {C}^n)$. However, they use only shears and overshears from formulas (1) and (2) which we would like to call *coordinate shears* and *coordinate overshears*. Our definition is more general. If one uses all LNDs instead of only coordinate shears, the question becomes more tricky. It is for example not clear, whether the group generated by flows of all overshears for all LNDs can be generated by the overshears for a countable number of LNDs. For $\mathbb {C}^2$, the group of algebraic automorphisms can be generated by all shears along the coordinate directions. Hence, it is clear that the problem has a positive answer for $\mathbb {C}^2$. In $\mathbb {C}^3$, the algebraic coordinate shears are not sufficient to generate the group of algebraic automorphisms by the result of Shestakov and Umirbaev. However, it is conjectured that a single algebraic automorphism of $\mathbb {C}^n$ together with the affine group generates the algebraic automorphism group of $\mathbb {C}^n$ (for $n\ge 4$ see for example Kanel-Belov 2018 Problem (3) in the last section).

If this is true, it would most likely give a solution to our next problem in case $X= \mathbb {C}^n$. In this context we ask the following question.

### Problem 6.5

Let *X* be a complex-affine manifold with the algebraic overshear density property. Is the group generated by flows of holomorphic overshear vector fields meagre in $\mathop {\textrm{Aut}_\textrm{1}}{X}$?

For a partial derivative $\Theta = \frac{\partial }{\partial z_j}$ in $\mathbb {C}^n$, it is immediate that $\ker \Theta$ is a ring of functions in $n-1$ variables. An important ingredient in the above-mentioned proof of Andersén and Lempert is to have a good growth estimate (in terms of *d*) for the vector space dimension of $\ker \Theta \cap \{ f \in \mathbb {C}[z_1, \dots , z_n] \,:\, \deg f \le d \}$ where $\deg$ is the total degree of *f*. This naturally leads to the following more general question for LNDs.

### Problem 6.6

Let *X* be a smooth affine variety over an algebraically closed field *k* of characteristic zero admitting a $\mathbb {G}_m$-action. Let $k[X] = \bigoplus _{j=0}^{\infty } B_j$ be the grading induced by this $\mathbb {G}_m$-action, and denote by $\deg$ the corresponding degree function. Let $\Theta$ be a locally nilpotent derivation on *k*[*X*]. Does the following hold?

$$\begin{aligned} \lim _{d \rightarrow \infty } \frac{\dim \{ f \in \ker \Theta :\, \deg f \le d \}}{\dim \{ f \in k[X] :\, \deg f \le d \}} = 0 \end{aligned}$$

Kaliman (2015) shows that an algebraic isomorphism of two affine subvarietes $X, Y \subset \mathbb {C}^n$ extends to a holomorphic automorphism of $\mathbb {C}^n$ if $n > \max (2 \dim X, \dim T X)$. The key ingredient in his proof is to use overshears of a suitable LND. More recently, an improved theorem was also given by Kaliman (2020).

### Problem 6.7

Let *Z* be an affine-algebraic manifold with the algebraic overshear density property. Let *X* and *Y* be algebraically isomorphic irreducible subvarieties of *Z*. Does every algebraic isomorphism $X \rightarrow Y$ extend to a holomorphic automorphism $Z \rightarrow Z$?

### Problem 6.8

Can all results about embeddings of open Riemann surfaces into $\mathbb {C}^2$ from the recent article of Alarcón and Forstnerič (2023) be generalized to embeddings into complex-affine surfaces with the algebraic overshear density property?
